# An Exploration of Evolution, Maturation, Expression and Function Relationships in Mir-23∼27∼24 Cluster

**DOI:** 10.1371/journal.pone.0106223

**Published:** 2014-08-26

**Authors:** Tingming Liang, JiaFeng Yu, Chang Liu, Li Guo

**Affiliations:** 1 Jiangsu Key Laboratory for Molecular and Medical Biotechnology, College of Life Science, Nanjing Normal University, Nanjing, Jiangsu, China; 2 Shandong Provincial Key Laboratory of Functional Macromolecular Biophysics, Institute of Biophysics, Dezhou University, Dezhou, Shandong, China; 3 Department of Epidemiology and Biostatistics, School of Public Health, Nanjing Medical University, Nanjing, Jiangsu, China; Huazhong University of Science and Technology, China

## Abstract

The study aims to explore the potential relationships of evolution, maturation, expression and function between homologous/clustered miRNAs. mir-23∼27∼24 gene cluster, including the two gene clusters (mir-23a and mir-23b) and the three miRNA gene families (mir-23, mir-27 and mir-24), was typically selected as an example. These related miRNAs show similar evolutionary patterns and various expression patterns. Most of them show consistent isomiR expression pattern, and the “switching” phenomenon can be found between different abundant isomiR species. These findings suggest that these sequence or location related miRNAs show the similar miRNA processing and maturation processes, and the robust selection of the most dominant isomiR exists in specific tissues. Functional analysis show that these miRNAs show similar distributions of enriched gene categories, suggesting the close functional prelateships via direct or indirect coordinate regulation in biological processes. The study reveals the close evolutionary, expression and functional relationships between related homologous/clustered miRNAs, which will further enrich miRNA studies and understand direct or indirect interactions between miRNAs.

## Introduction

MicroRNAs (miRNAs) are a class of non-coding RNAs (ncRNAs) with shorter size (∼22 nucleotides). They are vital to many cell functions via repressing target mRNAs at the post-transcriptional level [1]. miRNAs are generated from primary miRNAs (pri-miRNAs) and precursor miRNAs (pre-miRNAs) through cleavage of Drosha and Dicer [2], [3]. Typically, miRNA:miRNA* duplex is yielded by pri-miRNA and pre-miRNAs via cleavage process. miRNA is an active regulatory molecule, while miRNA* is ever thought as an inactive degraded strand [4]. However, increasing evidence indicates that the ever called passenger strand may also be abundantly expressed and play a biological role as a negative regulatory molecule [5]–[9]. The small ncRNA regulates target mRNAs by miRNA:mRNA interaction through complementarily binding of “seed sequences” of miRNA and 3′ untranslated region (3′ UTR) of mRNA.

miRNA is ever studied and predicted as a single sequence as well as annotated miRNA sequence in the miRBase database. However, recently, some studies have shown that miRNA locus can generate a series of sequence with heterogeneous 5′ and/or 3′ ends, and some are involved in 3′ post-transcriptional additional non-template nucleotides [10]–[22]. These physiological miRNA isoforms, also termed miRNA variants or isomiRs, have been concerned in miRNA study. Some isomiRs can be differentially loaded into Argonautes [16], [23], and isomiRs with 3′ addition are less prone to be degraded [17]. These miRNA isoforms are mainly derived from imprecise and alternative cleavage through pre-miRNA processing, and 3′ addition events [19]. Among these isomiRs, only several isomiRs are dominantly expressed despite multiple isomiRs can be detected [18], [22], [24], [29]. and most of them are 3′ isomiRs with the same 5′ ends and seed sequences [18], [24], [25]. The result implicates the potential dominant cleavage or cleavage bias through pre-miRNA processing. IsomiR expression patterns may be different across different miRNA loci, but they are always conserved in different samples, even across different tissues and animal species [18], [26]. Homologous and clustered miRNAs are prone to detect consistent isomiR expression profiles [27], although they may be differentially expressed with different expression levels [28]–[30]. Simultaneously, these sequence or location related miRNAs also have close evolutionary relationships and potential functional relationships [8], [31]–[33]. More miRNA gene clusters and families have been studied as potential biomarkers in diagnosis of human diseases, because they are believed as crucial regulator in multiple biological processes through contributing to the coding-non-coding RNA regulatory network.

In the present study, we attempted to discuss the potential relationships of evolution, maturation, expression and function between homologous/clustered miRNAs. Simultaneously, based on these results, the study aimed to track miRNA or isomiR maturation process between homologous/clustered miRNA loci. A specific example of mir-23∼24∼27 gene cluster was typically selected in the study. Specifically, the cluster includes miR-23a gene cluster (including mir-23a, mir-27a and mir-24-2 genes) and miR-23b cluster (including mir-23b, mir-27b and mir-24-1 genes). In human, the two miRNA gene clusters are located on chromosome 19(−) and chromosome 9(+), respectively, and both of them are reported as crucial miRNAs with important biological roles [34]–[38]. Antisense miRNA gene of mir-24, mir-3074 gene, can be detected in human, mouse and rat. The three miRNA gene families (mir-23, mir-27 and mir-24 gene families) are involved in the two gene clusters, and a total of five miRNAs can be yielded from these miRNA loci (the two miRNA genes, mir-24-1 and mir-24-2, can yield the same mature miR-24 sequence). Homologous miRNAs are located in different clusters, and these clustered miRNAs can be co-transcribed. Based on the homologous and/or clustered relationships and their important biological roles, the several related miRNAs are typically selected to perform the analysis to track miRNA or isomiR maturation process using evolutionary and expression analysis. The study can provide information for miRNA maturation, evolutionary, expression and functional relationships between sequence or location related miRNAs.

## Materials and Methods

### Source data

The pre-miRNAs, miRNA sequences and their annotations from different animal species were obtained from the miRBase database (Release 20.0, http://www.mirbase.org/) [39] (Figure S1). miRNA gene cluster was named based on close physical distances with mir-23a and mir-23b genes on the same chromosome, respectively (<10 kb). Although mir-3074 gene is identified as an antisense gene of mir-24 in several species, it is not involved in the analysis (the two sense and antisense miRNAs are also believed as clustered miRNAs).

miRNA expression data were collected from small RNA sequencing data in The Cancer Genome Atlas (TCGA) pilot project which is established by the NCI and NHGRI (https://tcga-data.nci.nih.gov/tcga/dataAccessMatrix.htm). Expression data of several specific miRNAs at the miRNA and isomiR levels, including miR-23a, miR-23b, miR-24, miR-27a and miR-27b, were further extracted using self-developed scripts. Small RNA sequencing data from eight human diseases and their normal tissues were analyzed in the study (Table 1).

**Table 1:** Sequencing datasets from the TCGA database are analyzed.

Disease	Source	TN	NT	Total
Breast cancer (BRCA)	TCGA	683	87	770
Clear cell carcinoma (KIRC)	TCGA	237	71	308
Colon and rectal adenocarcinoma (COAD, READ)	TCGA	471	16	487
Stomach adenocarcinoma (STAD)	TCGA	261	38	299
Lung adenocarcinoma (LUAD)	TCGA	436	46	482
Uterine corpus endometrial carcinoma (UCEC)	TCGA	383	21	404
Lung squamous cell carcinoma (LUSC)	TCGA	331	45	376
Thyroid carcinoma (THCA)	TCGA	507	59	566
Total		3,309	383	3,692

TN: Tumor, Matched Normal. NT: Normal, Matched Tumor.

### Evolutionary analysis

Pre-miRNA and miRNA sequences in different animal species were firstly aligned using Clustal X 2.0 software [40], and nucleotide divergence was then estimated with MEGA 5.10 software [41] and DnaSP 5.10.01 software [42] as miRNA population [43]. Nucleotide diversity (π) and haplotype diversity (Hd) in each miRNA were calculated using DnaSP 5.10.01. miR-#-5p, the ever passenger strand, were reported and annotated in some species, and other miR-#-5p sequences were predicted based on hsa-miR-#-5p using pre-miRNA sequences. Because the difference of miRNA sequences were not always involved in varied nucleotides, they were mainly derived from length difference during miRNA processing and maturation processes. Therefore, nucleotide diversity and haplotype diversity were estimated using consensus sequences with human miRNA sequences, and the difference, particular the length difference, was avoided in analysis.

Phylogenetic trees based on neighbor-net method [44] of miRNA genes (herein using pre-miRNA sequences) were reconstructed with SplitsTree 4.10 software [45] using Jukes-Cantor model. Further, evolutionary networks were reconstructed with Network 4.6.1.2 (http://www.fluxus-engineering.com/) using the median-joining (MJ) method. The parameter of the value of epsilon was set 10, and the generated MJ network was further optimized using Maximum Parsimony (MP) calculation.

### Expression and function analysis

The small RNA sequencing datasets in tumor and normal tissues were obtained from the TCGA (https://tcga-data.nci.nih.gov/tcga/dataAccessMatrix.htm) (Table 1). Based on relative expression rate, isomiR expression patterns in the miRNA locus were estimated using collected isomiR datasets from the TCGA (https://tcga-data.nci.nih.gov/tcga/dataAccessMatrix.htm). The expression patterns at the miRNA levels were assessed using collected miRNA datasets from the TCGA (https://tcga-data.nci.nih.gov/tcga/dataAccessMatrix.htm).

To find the potential functional relationships, predicted target mRNAs of these related miRNA gene families were obtained from the TargetScan program (http://www.targetscan.org/) (the cutoff of total context score was ≤ −0.30) [46], and they were further queried for Gene Ontology Enrichment categories using the CapitalBio Molecule Annotation System V4.0 (MAS, http://bioinfo.capitalbio.com/mas3/). Herein, the target mRNAs were predicted based on miRNA gene families, including homologous miRNAs and multiple isomiRs with the same seed sequences, and the main reason was that seed sequence of miRNA was the most crucial in miRNA-mRNA interaction.

### Statistical analysis

The relative expression rates of isomiRs were described using 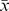 ± *sd* (mean ± standard deviation), and the expression levels of miRNAs were described using 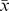 ± *se* (mean ± standard error) (because the expression of miRNAs were assessed using the original sequence counts). The difference between physical distance of miR-23a and miR-23b clusters was estimated using paired *t-*test. A *t-*test was used to estimate the difference between isomiR expression profiles from tumor and normal samples using all the isomiRs and the most dominant isomiR, respectively. If the *P* value is less than 0.05, and differences were considered statistically significant. In the study, all tests were conducted in Stata software (Version 11.0).

## Results

### Overview of the several related miRNAs

**Figure 1:**
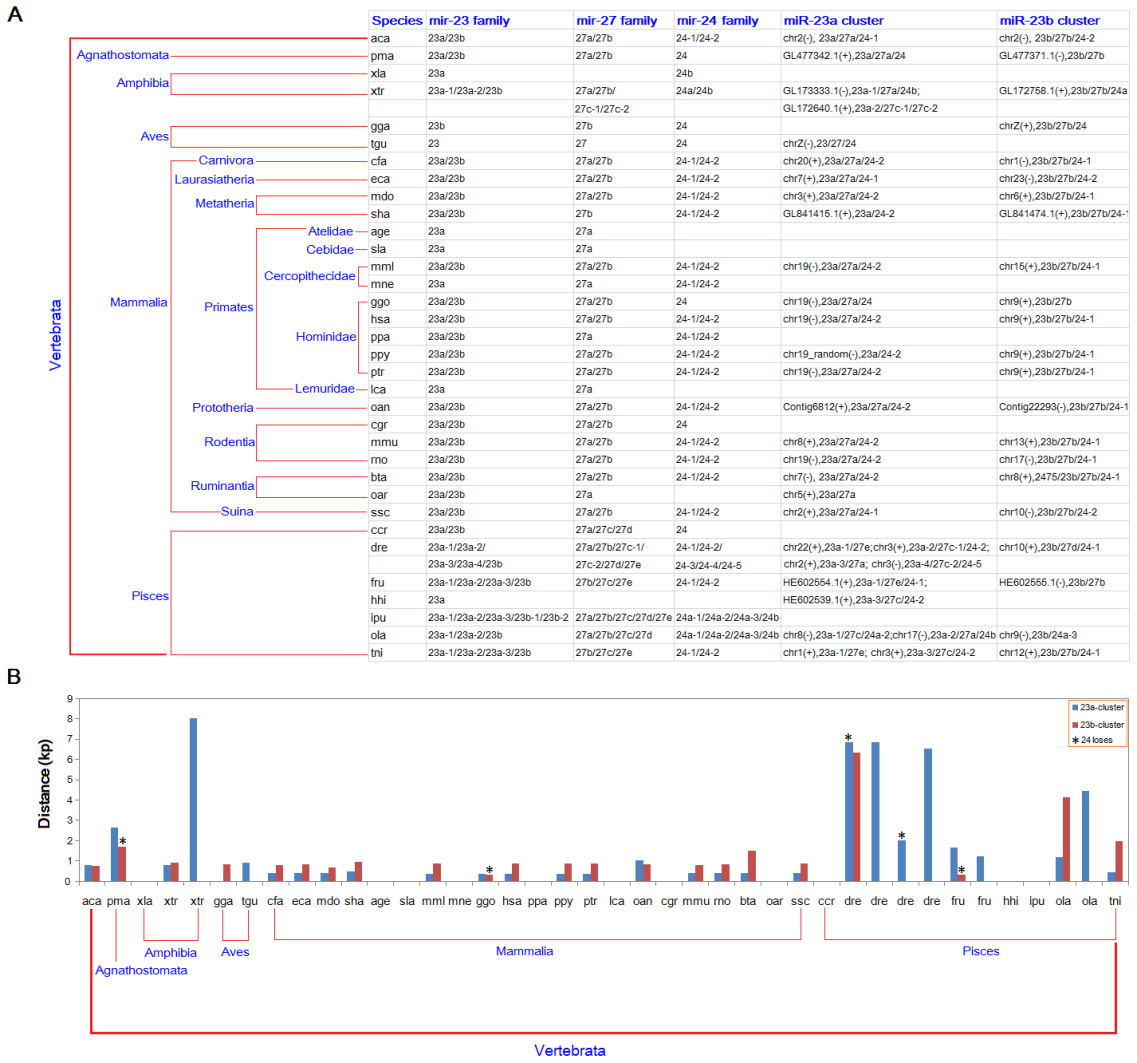
Evolutionary taxa, related miRNA members and physical distance of gene cluster in different animal species. (A) The three miRNA gene families have been detected in vertebrates. Their members are prone to be clustered on specific genomic region. Blank in columnn of gene family indicates that the miRNA members are not detected or predicted; blank in columnn of gene cluster indicates that no location annotation based on the current database. (B) Distribution of the physical distance between clustered miRNAs (miR-23a cluster and miR-23b cluster). The label of “24 loses” indicates that miR-24 is not detected in the species. As a terminus miRNA gene in gene cluster, the loss of miR-24 will influence the physical distance of the miRNA gene cluster.

mir-23, mir-27 and mir-24 gene families were mainly found in vertebrates (Figure 1A). According to the current database, they had been detected or predicted in most vertebrates. Numbers of homologous miRNA members might be diverse across different animal species, and the difference was mainly derived from multicopy pre-miRNAs, additional homologous miRNAs or deletion of members (Figure 1A). Compared to other vertebrates, fishes (Pisces) were prone to detect more homologous miRNAs in the three gene families (such as multiple pre-miRNAs for miR-23a and miR-24), and novel homologous miRNAs (such as miR-27c, 27d, 27e and 24b). miRNAs in the three gene families were always clustered on specific genomic region (Figure 1). Based on the close physical distance (<10 kb), miR-23a/miR-27a/miR-24 and miR-23b/miR-27b/miR-24 were composed of miRNA gene clusters. Interestingly, we found that the physical distances of the two miRNA gene clusters had significant difference (*t* = −3.4875, *P* = 0.003) (Figure 1B). In fishes, the distances were enlarged as well as more homologous miRNA members (Figure 1B).

### Similar evolutionary patterns

Generally, although there were 6 miRNA genes in mir-23∼24∼27 gene cluster, only 5 miRNAs were generated because multicopy pre-miRNAs for miR-24. The ever typical miRNAs were miR-#-3p, and all of them were well-conserved phylogenetically in vertebrates (Figure S1). Each miRNA had the same 5′ ends and “seed sequences” (nucleotides 2–8) across different animal species, and many species shared the common miRNA sequence based on the annotated miRNA sequences. Rare sequences were involved in varied nucleotides, and rare varied nucleotides were always detected in 3′ ends (Figure S1). Most different miRNA sequences were only detected difference in length distributions (various 3′ ends). The similar results could be detected between homologous miRNAs. Homologous miRNAs (such as miR-23a and miR-23, miR-27a and miR-27b) could be detected the common core sequence (more than 15 nucleotides) (Figure S1).

Simultaneously, another strand and loop sequence were also analyzed. Although the another strand was also well-conserved across vertebrates, they have high nucleotide diversity and haplotype diversity due to involved more varied nucleotides (Table S2). Compared to miR-#-5p and miR-#-3p sequences, the middle sequences, also termed loop sequences, were not conserved across different species (Table S2). For those multicopy pre-miRNAs, although they could yield the same miRNAs, they might be involved in varied nucleotides, including insertions/deletions, especially in the loop sequences. Evolutionary networks of miRNAs showed that miR-#-5p and miR-#-3p had different evolutionary patterns, and miR-#-5p sequences were prone to be involved in complex networks with more median vectors (Figure S2). Larger genetic distances could be detected between miR-#-5p sequences, although miR-#-3p sequences were highly conserved across different animal species. Phylogenetic trees of pre-miRNAs also showed various patterns, although miRNA genes were homologous or clustered together (Figure S3).

### Expression patterns and functional enrichment analysis

**Figure 2:**
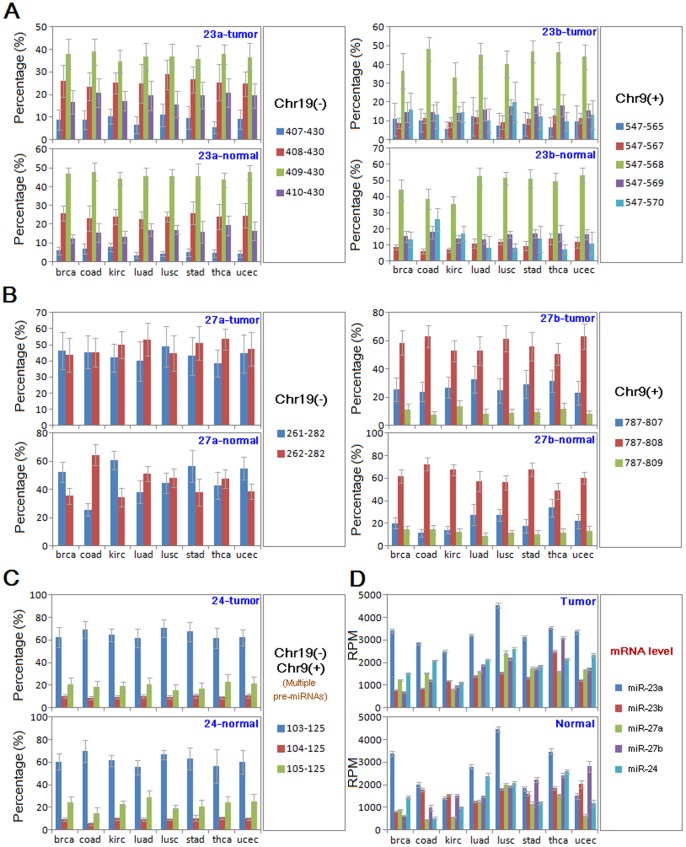
Expression patterns at the miRNA/isomiR levels. Expression patterns of miRNAs are presented here at the miRNA/isomiR levels in the 8 tumor samples and their normal samples. (A) expression of miR-23a and miR-23b at the isomiR levels; (B) expression of miR-27a and miR-27b at the isomiR levels; (C) expression of miR-24 at the isomiR levels; (D) expression of the five miRNAs at the miRNA levels.

Using public small RNA sequencing datasets, we found that some of these related miRNAs showed similar isomiR expression, although rates of dominant isomiRs were diverse (Figure 2). Among of these related miRNAs, we found that most of miRNA loci only yielded a kind of quite dominant isomiR, while miR-27a locus was detected two dominant isomiRs with similar expression (Figure 2). IsomiR expression patterns were always stable across different tumor/normal samples, but some miRNAs showed inconsistent expression. For example, isomiRs from miR-27a indicated “switching” between the two dominant isomiRs across different samples (Figure 2B). However, similar phenomenon was not detected in isomiRs from miR-27b, although it was homologous miRNA with miR-27a.

Compared to relative stable isomiR expression patterns, these related miRNAs may show diverse expression levels across different tumor/normal samples, but the similar expression tendency could be detected between the same tissues (including tumor and normal samples, unless the miRNA was abnormally expressed in tumor samples). No significant difference of isomiR expression could be detected between tumor and normal samples (*P*>0.05, Table S1). However, based on the most dominant isomiR, miR-23a and miR-24 showed inconsistent expression (miR-23a, *P*<0.0001; miR-24, *P*<0.01, Table S1). These related miRNAs also showed similar expression between tumor and normal samples based on the dominant isomiR (*P*>0.05, Table S2).

**Figure 3:**
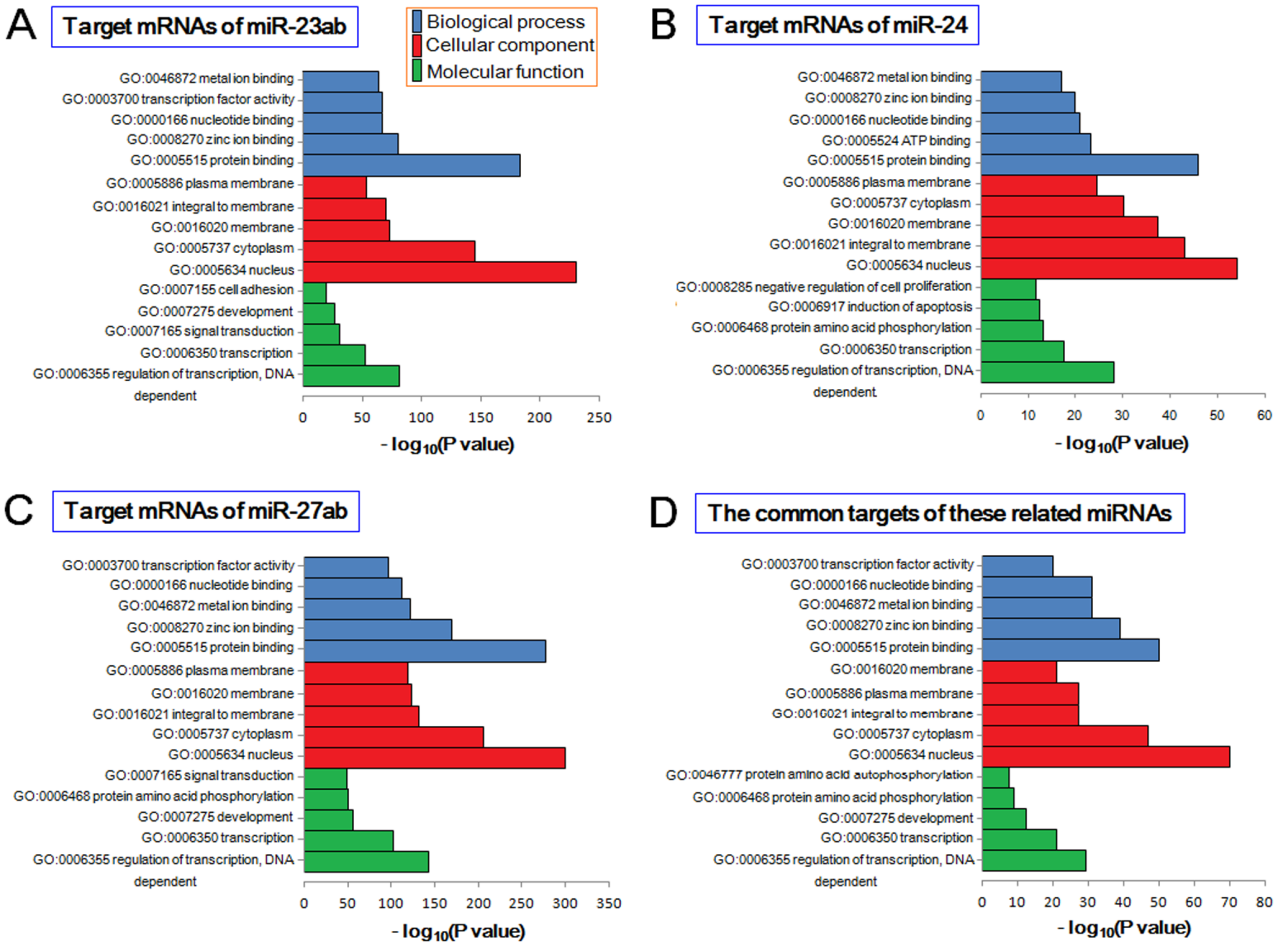
The top 5 gene categories based on target mRNAs of related miRNAs. The top 5 gene categories, including biological process, cellular component and molecular function, are presented according to target mRNAs of (A) miR-23ab, (B) miR-24, (C) miR-27ab and (D) the common target mRNAs of the three miRNA gene families.

Gene categories of the three related miRNA gene families showed similar distributions with the same most significantly enriched categories (Figure 3 and Table S3). Although they had different seed sequences and target mRNAs, these related miRNAs contributed to the same or similar biological process, cellular component and molecular function (Figure 3 and Table S3). Further KEGG pathway enrichment analysis also showed similar enriched pathways between the three miRNA families (Figure S4 and Table S3). Although the three gene families had different seed sequences, the common target mRNAs were also collected and analyzed. The main reason may be derived from different binding regions in the same target mRNA.

## Discussion

The three miRNA gene families have been widely studied, especially as potential biomarker in human diseases [47]–[50]. They are prone to locate in gene cluster with close physical distance on chromosomes, and the similar miRNA members and distance distributions can be found in most vertebrates (Figure 1). The related members in the three miRNA gene families show similar evolutionary patterns, although they also indicate a slight difference (Table S2, Figures S1, S2 and S3). Specifically, mir-24 genes are not evolved the two homologous miRNAs as well as mir-23 and mir-24 genes, despite the two multicopy pre-miRNAs of miR-24 can also be detected in most vertebrates (except for fishes, Table 2 and Figure 1). Phylogenetic trees using pre-miRNAs show diverse patterns, which is mainly derived from nucleotide divergence between homologous miRNA genes and multicopy pre-miRNAs, particularly in loop and non-dominant miRNA sequences in fishes (Table 2 and Figure S3). In fishes (Pisces), both miR-23a/b and miR-24 have been detected multicopy pre-miRNAs, while miR-27 are prone to detect homologous members. The interesting results implicate that the three miRNA gene families may be further duplicated and extended in fishes with various levels of nucleotide divergence as well as larger physical distances. Simultaneously, miR-23a/b and miR-24 sequences are well-conserved, and the duplicated miRNA genes are rarely involved in varied nucleotides in miRNAs. However, duplicated miR-27 genes are involved in varied nucleotides in miRNA regions, which therefore leads to homologous miRNA members with higher sequence similarity (miR-27a/b/c/d/e). In contrast to the well-conserved miR-23 and miR-24 gene families, the rapid evolution process of miR-27 family may imply the potential functional and evolutionary pressures in fishes. Moreover, the phenomenon of multicopy pre-miRNAs or multiple homologous miRNAs are prone to detect in fishes according to analysis of other miRNAs, which may be derived from genome duplication [51]. The duplication event further complicates the miRNA world and the coding-non-coding RNA regulatory network.

**Table 2:** Nucleotide diversity (π) and haplotype diversity (Hd) of miRNA populations.

miRNA	miR-#-3p (dominant)		Loop sequences		miR-#-5p	
	π	Hd	π	Hd	π	Hd
miR-23a	0.002±0.002	0.047±0.044	0.618±0.030	0.939±0.018	0.157±0.008	0.695±0.051
miR-23b	0.003±0.003	0.071±0.065	0.126±0.033	0.706±0.090	0.041±0.011	0.537±0.109
miR-23	0.025±0.002	0.501±0.028				
miR-27a	0.004±0.003	0.074±0.067	0.381±0.058	0.923±0.038	0.116±0.029	0.561±0.114
miR-27b	0.004±0.004	0.083±0.075	0.230±0.046	0.859±0.052	0.041±0.027	0.239±0.113
miR-27	0.414±0.013	0.547±0.030				
miR-24	0.002±0.002	0.037±0.035	0.464±0.028	0.908±0.028	0.130±0.016	0.760±0.039

The consensus sequences were estimated based on human miRNA or loop sequences. miR-23, including miR-23a and miR-23b, was estimated π and Hd based on the dominant miRNA sequences (the loop sequences and another strands would be involved in larger levels of nucleotide divergence).

Expression patterns at the miRNA and isomiR levels of these related miRNAs are further analyzed in different tumor samples using public small RNA datasets. For a specific miRNA locus, similar isomiR expression patterns indicate the stable miRNA maturation and processing mechanisms (Table S1). Although sequence or physical related miRNA loci are prone to show similar isomiR expression profiles [29], miR-27a show specific expression patterns with the two similar dominant isomiRs (Table S1, Table S2 and Figure 3). The two isomiRs can be switched in different samples, which suggests that the potential switching phenomenon in isomiR expression profiles. Switching events have been found in expression or changes of ratio between the two arms of miR-#-5p and miR-#-3p, and herein we also call switching event in the dynamic selection of dominant isomiR. Indeed, the two isomiRs have the same 5′ ends and seed sequences, and they only diverge in 3′ end (Figure 1). The interesting switching event in selection of dominant isomiRs supports that multiple isomiRs provide the opportunity to select the most appropriate dominant isomiR [25]. Although most isomiRs from an miRNA locus have the same seed sequences and targets, the divergence of length and 3′ ends may also play unclear roles in the miRNA world as well as homologous miRNAs and various miRNA sequences in different animal species. As homologous miRNAs, such as miR-23a/23b and miR-27a/27b, they are prone to have the same seed sequences and target mRNAs, although they have different sequence with varied nucleotides. Simultaneously, some miRNA sequences in different animal species may be involved in heterogeneous sequences with varied nucleotides or lengths, although most of them are well-conserved, particularly in the seed sequences. However, we poorly understand the potential effects of diverged nucleotides in other positions (except for seed sequences) and length distributions in miRNA/isomiR in the evolutionary and functional miRNA studies.

Functional analysis indicate that the three related miRNA gene families contribute to similar biological pathways, despite they have different seed sequences and predicted target mRNAs (Figures 3, S4 and Table S3). The surprising similar functions implicate direct or indirect coordinate regulation patterns between these sequence or location related miRNAs. *In vivo*, these miRNAs may show diverse expression patterns [27]–[30], and a major reason may be regulated expression as candidate small RNA regulatory molecules. The dynamic expression of related miRNAs may flexible adapt to functional need.

Taken together, the study using mir∼23∼27∼24 provides more evolution, maturation, expression and function relationships between homologous/cluster miRNAs. Similar evolutionary patterns, miRNA maturation and processing processes and functional relationships implicate the “functional groups” of miRNAs/isomiRs in regulatory networks. Simultaneously, the close sequence and location relationships also provide the opportunity to direct or indirect coordinate regulatory pattern between different miRNAs. These results are also adaptable to other related homologous or clustered miRNAs, and these evolutionary, expression and functional relationships will further enrich miRNA studies and understand direct or indirect interactions between miRNAs.

## Supporting Information

Figure S1 **The five related miRNAs are well-conserved in vertebrates.** All of them are only involved in difference in 3′ ends, and less are detected varied nucleotides. Homologous miRNAs are also detected the common core sequences.
(PPT)

Figure S2 **Evolutionary networks of miRNAs.** (A) Evolutionary networks of miRNA members in miR-23 gene family. (B) Evolutionary networks of miRNA members in miR-27 gene family. (C) Evolutionary network of miR-24-5p. Each miRNA (including miR-#-5p and miR-#-3p) was reconstructed the evolutionary network. miR-23a-3p, miR-27a-3p and miR-24-3p could not be reconstructed due to conserved sequences (less than 3 different sequences). However, networks of miR-23-3p (including miR-23a-3p and miR-23b-3p) and miR-27-3p (including miR-27a-3p and miR-27b-3p) were reconstructed. The size of the circle indicates that the miRNA sequence is shared by the number of species. The purple circle indicates miR-#-3p, the yellow circle indicates miR-#-5p, and the red circle indicates the mediate vector that is hypothesized miRNA sequence.
(PPT)

Figure S3 **Phylogenetic trees of miRNA genes.** (A) Phylogenetic trees of homologous mir-23a and mir-23b. (B) Phylogenetic trees of homologous mir-27a and mir-27b. (C) Phylogenetic tree of mir-24.
(PPT)

Figure S4 **The top 20 most enriched KEGG pathways of target mRNAs of related miRNAs.** KEGG pathways of (A) miR-23ab, (B) miR-24 and (C) miR-27ab are presented here according to target mRNAs. (D) indicates the top 20 most enriched KEGG pathways of the common target mRNAs of the three miRNA gene families.
(PPT)

Table S1 **The statistical analysis of related miRNAs between tumor and normal samples.**
(DOC)

Table S2 **The statistical analysis of miRNAs based on the most dominant isomiR.**
(DOC)

Table S3 **Functional enrichment results based on human miRNAs.**
(XLS)
