# Supplementary figures and images for: An Exploration of Evolution, Maturation, Expression and Function Relationships in Mir-23∼27∼24 Cluster
Source: PLoS One. 2014 Aug 26;9(8):e106223. doi: 10.1371/journal.pone.0106223 (PMC4144971; doi:10.1371/journal.pone.0106223)

## Slide 1
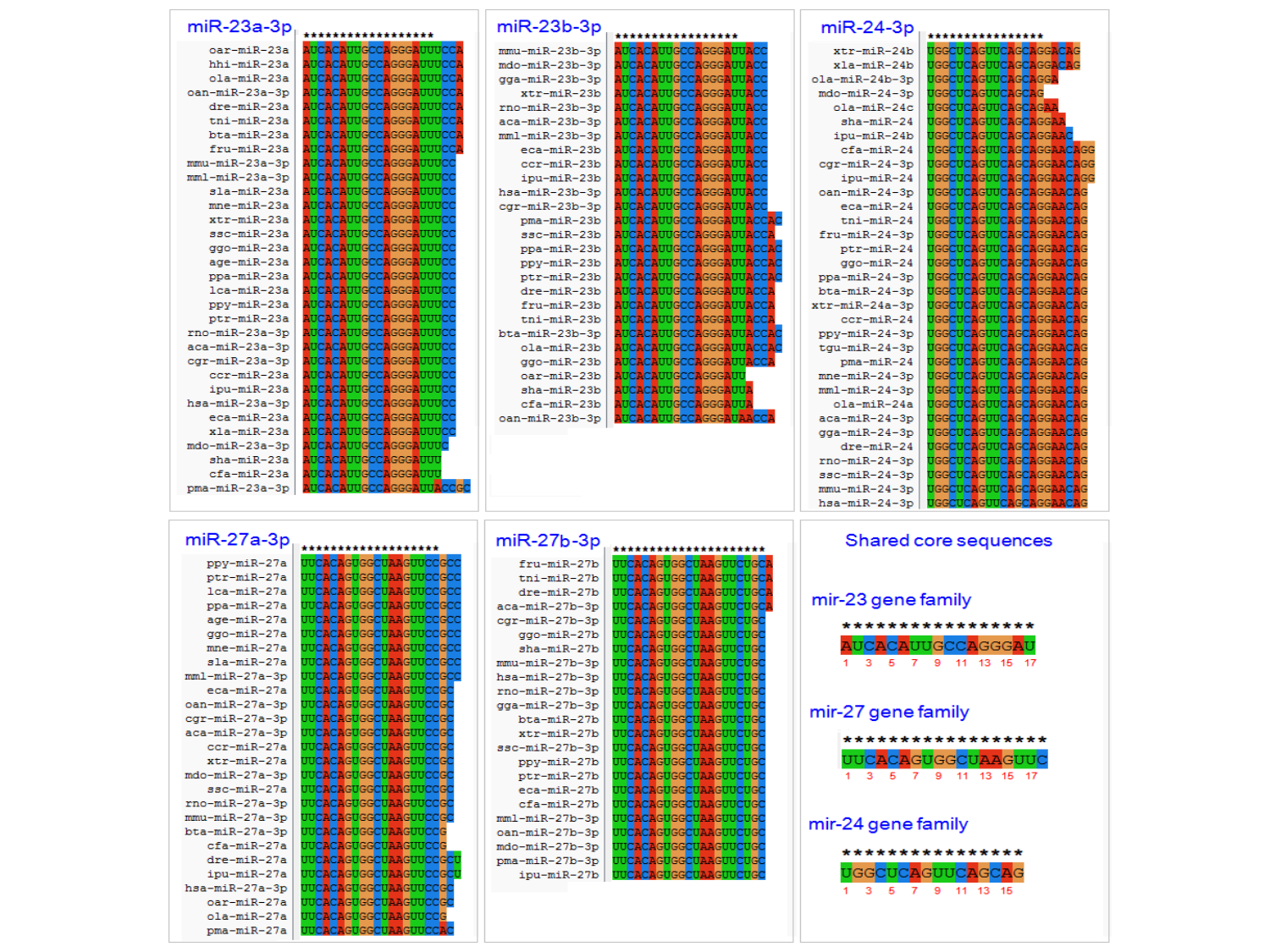

Supplement: Figure S1 — The five related miRNAs are well-conserved in vertebrates. All of them are only involved in difference in 3′ ends, and less are detected varied nucleotides. Homologous miRNAs are also detected the common core sequences. (PPT) [file pone.0106223.s001.ppt]

## Slide 1
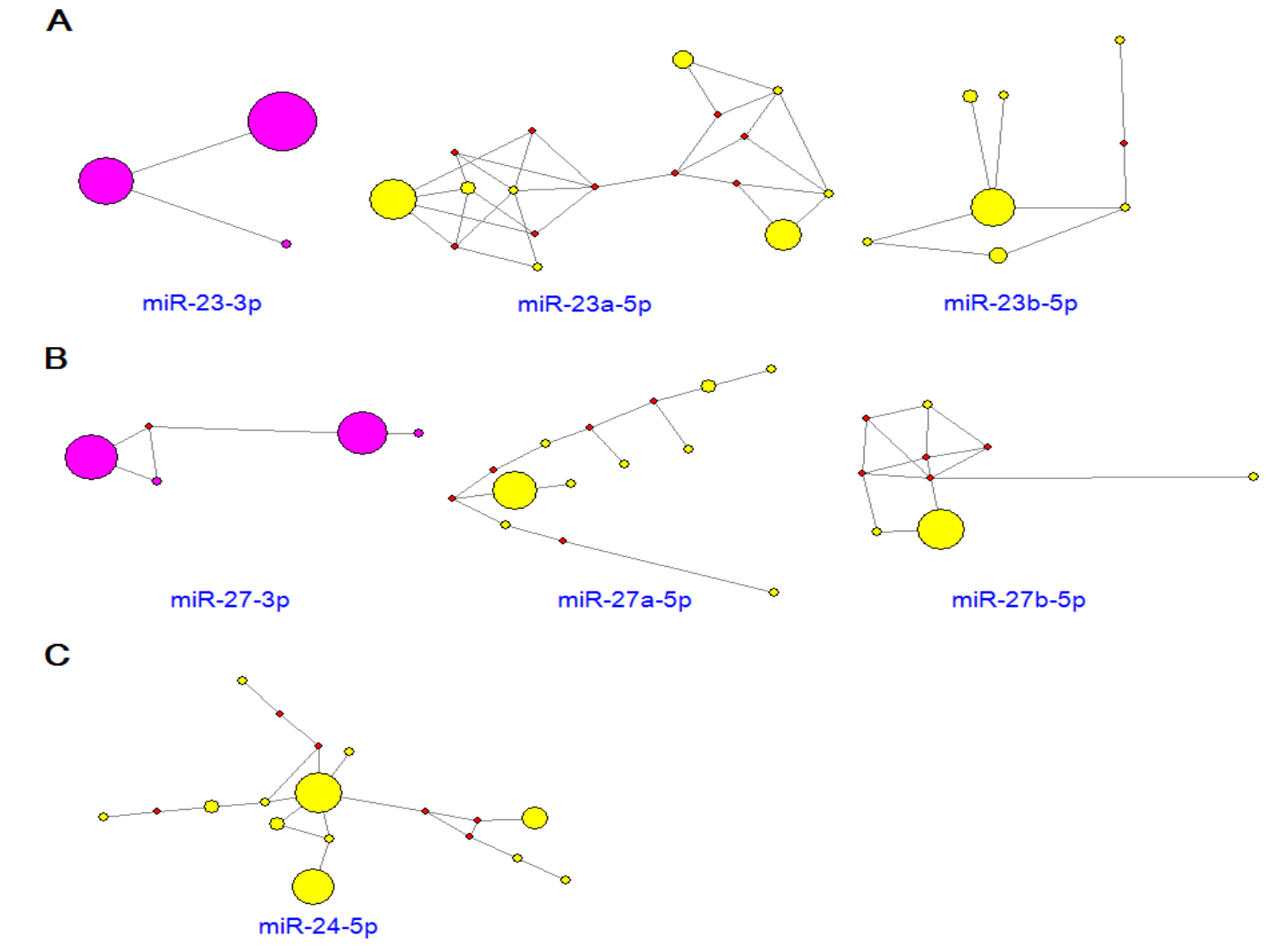

Supplement: Figure S2 — Evolutionary networks of miRNAs. (A) Evolutionary networks of miRNA members in miR-23 gene family. (B) Evolutionary networks of miRNA members in miR-27 gene family. (C) Evolutionary network of miR-24-5p. Each miRNA (including miR-#-5p and miR-#-3p) was reconstructed the evolutionary network. miR-23a-3p, miR-27a-3p and miR-24-3p could not be reconstructed due to conserved sequences (less than 3 different sequences). However, networks of miR-23-3p (including miR-23a-3p and miR-23b-3p) and miR-27-3p (including miR-27a-3p and miR-27b-3p) were reconstructed. The size of the circle indicates that the miRNA sequence is shared by the number of species. The purple circle indicates miR-#-3p, the yellow circle indicates miR-#-5p, and the red circle indicates the mediate vector that is hypothesized miRNA sequence. (PPT) [file pone.0106223.s002.ppt]

## Slide 1
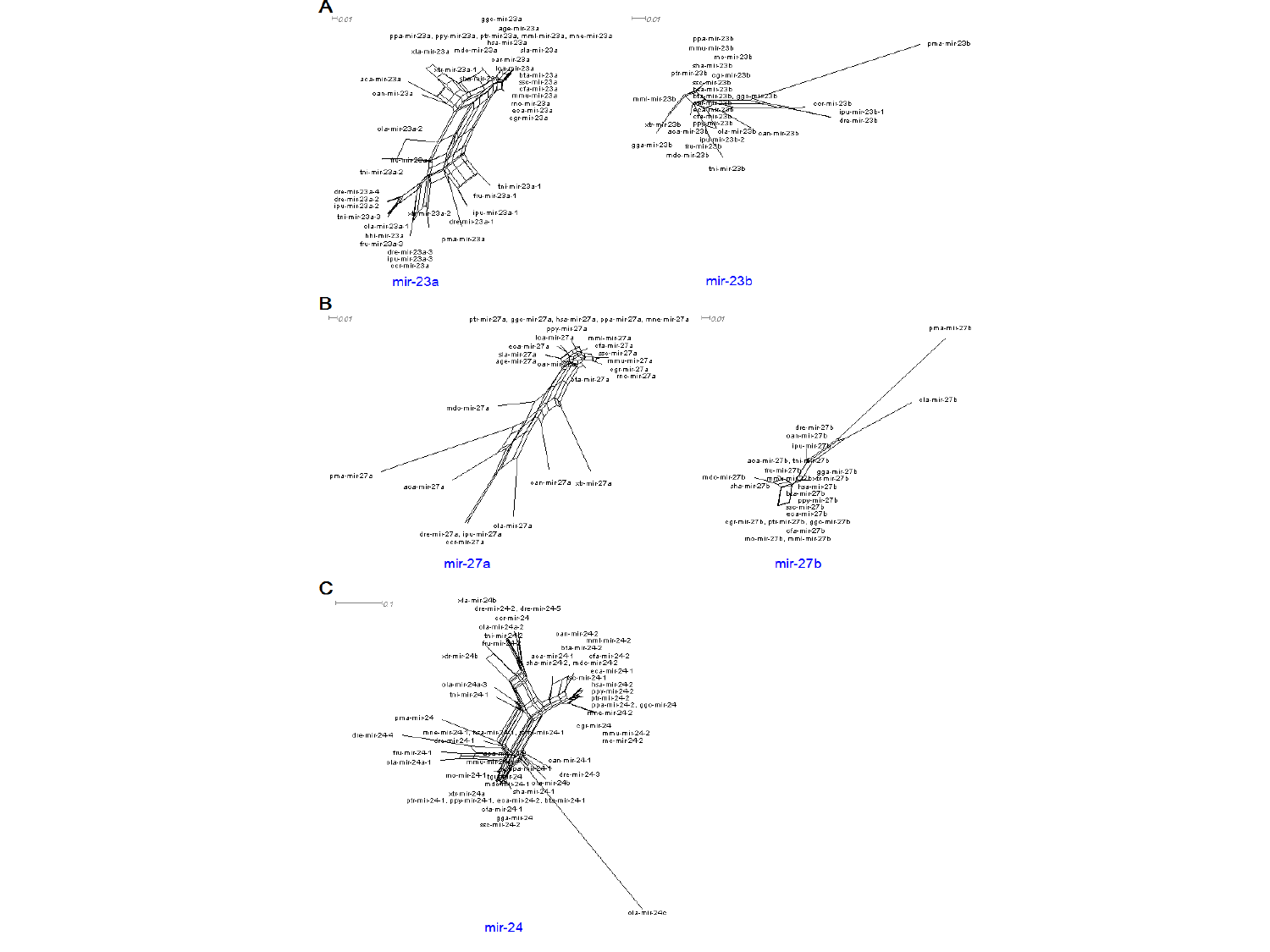

Supplement: Figure S3 — Phylogenetic trees of miRNA genes. (A) Phylogenetic trees of homologous mir-23a and mir-23b. (B) Phylogenetic trees of homologous mir-27a and mir-27b. (C) Phylogenetic tree of mir-24. (PPT) [file pone.0106223.s003.ppt]

## Slide 1
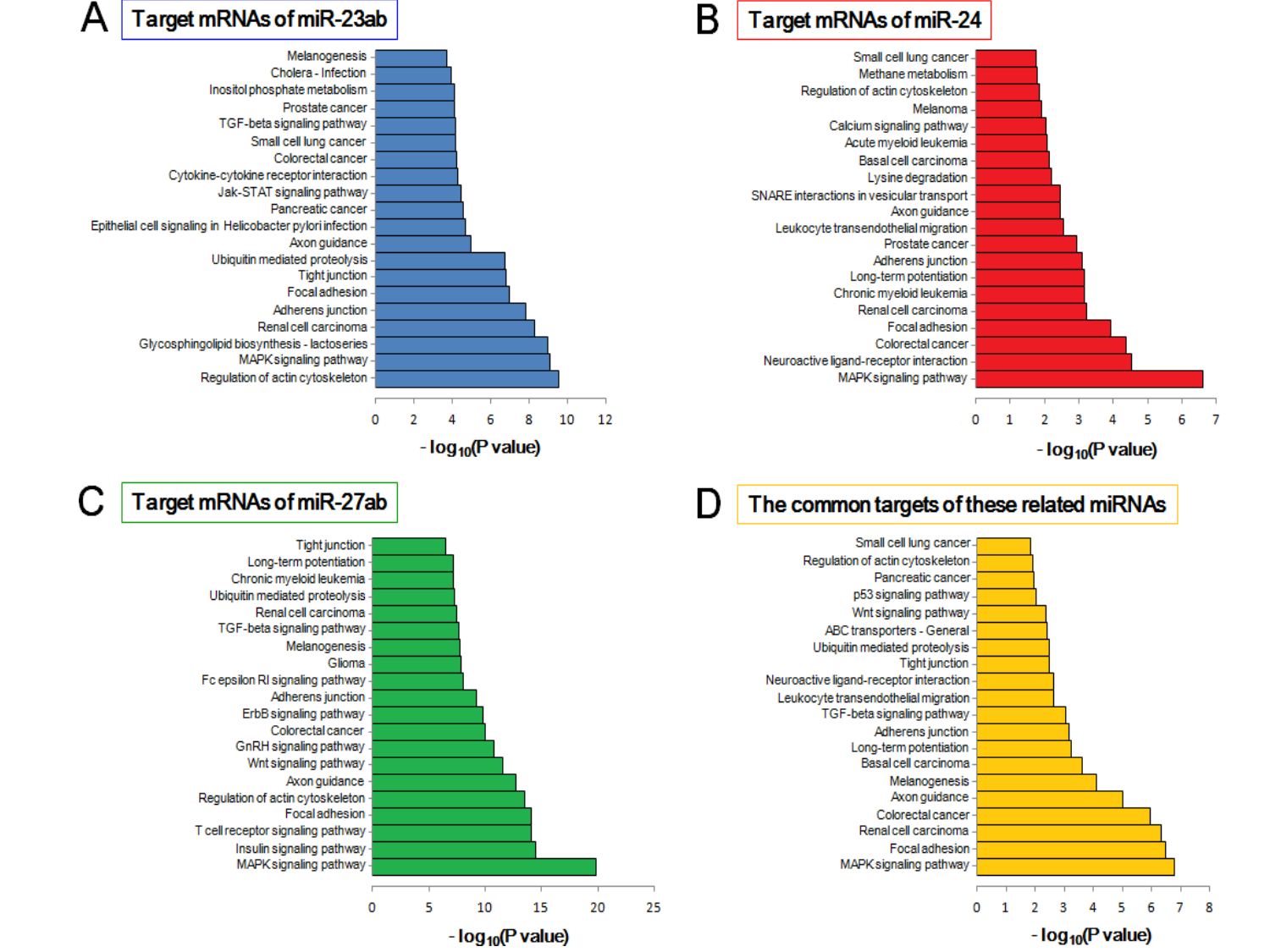

Supplement: Figure S4 — The top 20 most enriched KEGG pathways of target mRNAs of related miRNAs. KEGG pathways of (A) miR-23ab, (B) miR-24 and (C) miR-27ab are presented here according to target mRNAs. (D) indicates the top 20 most enriched KEGG pathways of the common target mRNAs of the three miRNA gene families. (PPT) [file pone.0106223.s004.ppt]
