# Supplementary material for: An Exploration of Evolution, Maturation, Expression and Function Relationships in Mir-23∼27∼24 Cluster
Source: PLoS One. 2014 Aug 26;9(8):e106223. doi: 10.1371/journal.pone.0106223 (PMC4144971; doi:10.1371/journal.pone.0106223)
Supplement: Table S1 — The statistical analysis of related miRNAs between tumor and normal samples. (DOC) [file pone.0106223.s005.doc]

**Table S1. The statistical analysis of related miRNAs between tumor and normal samples.**

|  | **Tumor and normal** | | **Tumor and normal** | |
| --- | --- | --- | --- | --- |
|  | *t (all isomiRs)* | *P (all isomiRs)* | *t* (the most isomiR) | *P* (the most isomiR) |
| miR-23a | -0.3814 | 0.7055 | -16.3952 | 0.0000 |
| miR-23b | -0.7377 | 0.4662 | -1.9359 | 0.0941 |
| miR-27a | 0.0676 | 0.9470 | 0.9696 (-0.7928)* | 0.3645 (0.4539) |
| miR-27b | -0.2470 | 0.8071 | -1.6119 | 0.1510 |
| miR-24 | 0.2416 | 0.8112 | 3.8773 | 0.0061 |

Statistical analysis were analyzed based on all isomiRs and the most dominant isomiR, respectively. * indicates that both the two kind of isomiRs of miR-27a are dominantly expressed with similar expression levels, and they are simultaneously estimated.
