# Supplementary material for: An Exploration of Evolution, Maturation, Expression and Function Relationships in Mir-23∼27∼24 Cluster
Source: PLoS One. 2014 Aug 26;9(8):e106223. doi: 10.1371/journal.pone.0106223 (PMC4144971; doi:10.1371/journal.pone.0106223)
Supplement: Table S2 — The statistical analysis of miRNAs based on the most dominant isomiR. (DOC) [file pone.0106223.s006.doc]

**Table S2. The statistical analysis of miRNAs based on the most dominant isomiR.**

| **Sample** | ***t* and *P* values** | **Sample** | ***t* and *P* values** |
| --- | --- | --- | --- |
| brca | *t* = -2.4982, *P* = 0.0669 | lusc | *t* = -0.3384, *P* = 0.7520 |
| coad | *t* = 0.3524, *P* = 0.7423 | stad | *t* = -2.2297, *P* = 0.0896 |
| kirc | *t* = -2.1162, *P* = 0.1018 | thca | *t* = -0.6728, *P* = 0.5379 |
| luad | *t* = -0.8609, *P* = 0.4379 | ucec | *t* = -1.6838, *P* = 0.1675 |
